# Assessment of dental pulp response to caries via MR *T*_*2*_ mapping and histological analysis

**DOI:** 10.1186/s12903-024-04165-1

**Published:** 2024-04-06

**Authors:** Ana Tenyi, Aleksandra Milutinović, Jernej Vidmar, Igor Serša, Ksenija Cankar

**Affiliations:** 1 Medical Faculty, Department of dental diseases and normal dental morphology, Hrvatski trg 6, University of Ljubljana, Ljubljana, Slovenia; 2 Medical Faculty, Institute of histology and embryology, University of Ljubljana, Ljubljana, Slovenia; 3 Institute of Radiology, University Medical Center Ljubljana, Ljubljana, Slovenia; 4 Medical Faculty, Institute of Physiology, University of Ljubljana, Ljubljana, Slovenia; 5 Jožef Stefan Institute, Ljubljana, Slovenia

**Keywords:** Magnetic resonance imaging, Dental pulp, Dental caries, Inflammation, Histology

## Abstract

The aim of our study was to assess the correlation between *T*_*2*_ relaxation times and their variability with the histopathological results of the same teeth in relation to caries progression. Materials and methods: 52 extracted permanent premolars were included in the study. Prior to extractions, patients underwent magnetic resonance imaging (MRI) scanning and teeth were evaluated using ICDAS classification. Pulps of extracted teeth were histologically analysed. Results: MRI *T*_*2*_ relaxation times (ms) were 111,9 ± 11.2 for ICDAS 0, 132.3 ± 18.5* for ICDAS 1, 124.6 ± 14.8 for ICDAS 2 and 112. 6 ± 18.2 for ICDAS 3 group (*p* = *0,013*). A positive correlation was observed between MRI *T*_*2*_ relaxation times and macrophage and T lymphocyte density in healthy teeth. There was a positive correlation between vascular density and *T*_*2*_ relaxation times of dental pulp in teeth with ICDAS score 1. A negative correlation was found between *T*_*2*_ relaxation times and macrophage density. There was a positive correlation between *T*_*2*_ relaxation time variability and macrophage and T lymphocyte density in teeth with ICDAS score 2. In teeth with ICDAS score 3, a positive correlation between *T*_*2*_ relaxation times and *T*_*2*_ relaxation time variability and lymphocyte B density was found. Conclusion: The results of our study confirm the applicability of MRI in evaluation of the true condition of the pulp tissue. Clinical relevance: With the high correlation to histological validation, MRI method serves as a promising imaging implement in the field of general dentistry and endodontics.

## Introduction

The degree of pulpal response caused by detrimental factors in the oral cavity environment is largely dependent on its potential to resist cariogenic bacterial interactions on one hand and defensive mechanisms resisting trauma of the hard dental tissues on the other [1]. Dense vascular system of the pulp tissue represents a crucial role in the maintenance of the pulpal homeostasis and offers a dynamic response to any harmful events. Microvascular reactions include modifying the local capillary filtration rate, adjusting the immune and inflammatory tissue processes and stimulation of angiogenesis [2].

Next to the vascular changes, local injury of the pulp tissue activates the inflammatory cell migration as well. Soon after the trauma of hard dental tissues or onset of the infection due to caries progression, several mediators induce transition of the neutrophils, monocytes, T lymphocytes and B lymphocytes from the vascular system into the tissue [3]. They have multifactorial role which includes bacterial destruction, cell residues disposal, antigen – representing role, reparation and restoration of the pulp tissue, promoting stimulation of angiogenesis and proliferation of the fibroblasts [4]. In a healthy pulp, B lymphocytes are uncommon compared to other inflammatory cells and infiltrate tissue in a later phase, after inflammation has already been evolved [5].

With the aim of the acquisition of the three–dimensional images of dental tissues, most commonly used imaging methods in dentistry include panoramic X–rays and cone beam computed tomography (CBCT). Both methods utilize ionizing radiation as a tool for contrast gaining, which carries the health risks as well as an inability to visualize soft tissues, such as the dental pulp. In contrast, magnetic resonance imaging (MRI) can also serve as a noninvasive diagnostic tool for assessment of dental tissues, especially of the pulp, since it enables the optimization of contrast among various soft tissues based on the values of *T*_*1*_ and *T*_*2*_ relaxation times of various tissues and organs [6–8]. *T*_*2*_ mapping is an advanced MRI technique used to calculate the *T*_*2*_ relaxation times of the specific tissues and displaying them voxel-vice on a parametric map. The *T*_*2*_ relaxation times reflect water content in the respective tissue and are nowadays mainly used for the evaluation of cardiac edema, e.g., in myocardial inflammation or infarction, as well as to describe the composition of tissues in other pathologies [9]. Over the recent years, MRI technology has improved diagnostic potential in the several fields of head, oral cavity and teeth pathology treatment, inlucluding oral and maxillofacial surgery [10, 11], periodontology [12] and general dentistry [13]. Among other novel treatment modalities in dentistry [14], MRI serves as an excellent tool to maximize diagnostic certainty which improves treatment outcomes. *T*_*2*_ mapping is proving to be useful for non-invasive evaluation of caries progression assessed with ICDAS classification standard [8]. Consequently, it enables good differentiation among different stages of soft tissue inflammation without the need of contrast medium and would be an appropriate method for the early detection of pulpal response to carious lesion [8, 15].

It is important to validate this new MRI method for the evaluation of dental pulp response to caries progression before its use in clinical practice. In medicine, the golden standard is histopathology which is used to validate the presence and type of a particular disease.

The aim of our clinical/histological study was to assess the correlation between *T*_*2*_ relaxation times and their variability with the histopathological results of the same teeth in relation to progression of carious lesion using ICDAS coding assessment.

## Materials and methods

### Ethical statement

The Slovenian national medical ethics committee approved the study under the protocol number 0120–415/2020/6. All invited participants received prior information and signed the informed consent form.

### Participants, MRI protocol, tooth extraction in preparation for histological analysis

#### Patients

Fifty-two permanent upper and lower premolars of fourteen patients, aged from 12 to 20 years, were included in the study. The teeth were scheduled for extraction due to orthodontic indication of teeth crowding. Prior to extractions, patients were scheduled for clinical examination and assessment of carious lesions on all tooth surfaces using ICDAS classification [16]. Carries assessments were performed by a single investigator, as described in previous studies [8]. According to their corresponding ICDAS score, teeth were divided into four groups. ***Group A***: intact teeth with ICDAS score 0, **group B**: initial carious lesion with ICDAS score 1, ***group C***: initial carious lesion with ICDAS score 2, and ***group D***: carious lesion with visible and palpable enamel cavitation, ICDAS score 3.

#### MR Image Acquisition

MR imaging was performed on a 3-T whole-body MRI system (TX Achieva, Philips, Netherlands) with a maximal gradient strength of 80 mT/m and with the use of a 32-channel receive head coil in all patients on the same day as the clinical examination. All patients were able to perform routine daily activities prior to each examination. The MR protocol consisted of a set of moderate resolution images to localize the dental pulp anatomy first [8]. For *T*_2_ mapping, multi-echo-spin-echo (MESE) *T*_*2*_-weighted sequence in sagittal slices and with a field of view that covered most of the pulp chamber was employed [8]. The parameters of the multi-echo-spin-echo (MESE) sequence were: TR/TE, 2000/15 ms as well as 30, 45, 60, 75, 90 ms; field of view 160 × 160 mm^2^; slice thickness 2.5 mm; image acquisition/reconstruction matrix 380 × 311/560 × 560; acquisition/reconstruction voxel size 0.42 × 0.51 × 2.5/0.29 × 0.29 × 2.5 mm^3^; three slices; gap 0.25; number of signal averages 1; no signal acquisition acceleration; and acquisition time for all 6 echoes was equal to 10 min 24 s [8].

#### MR Data Analysis

**Fig. 1:**
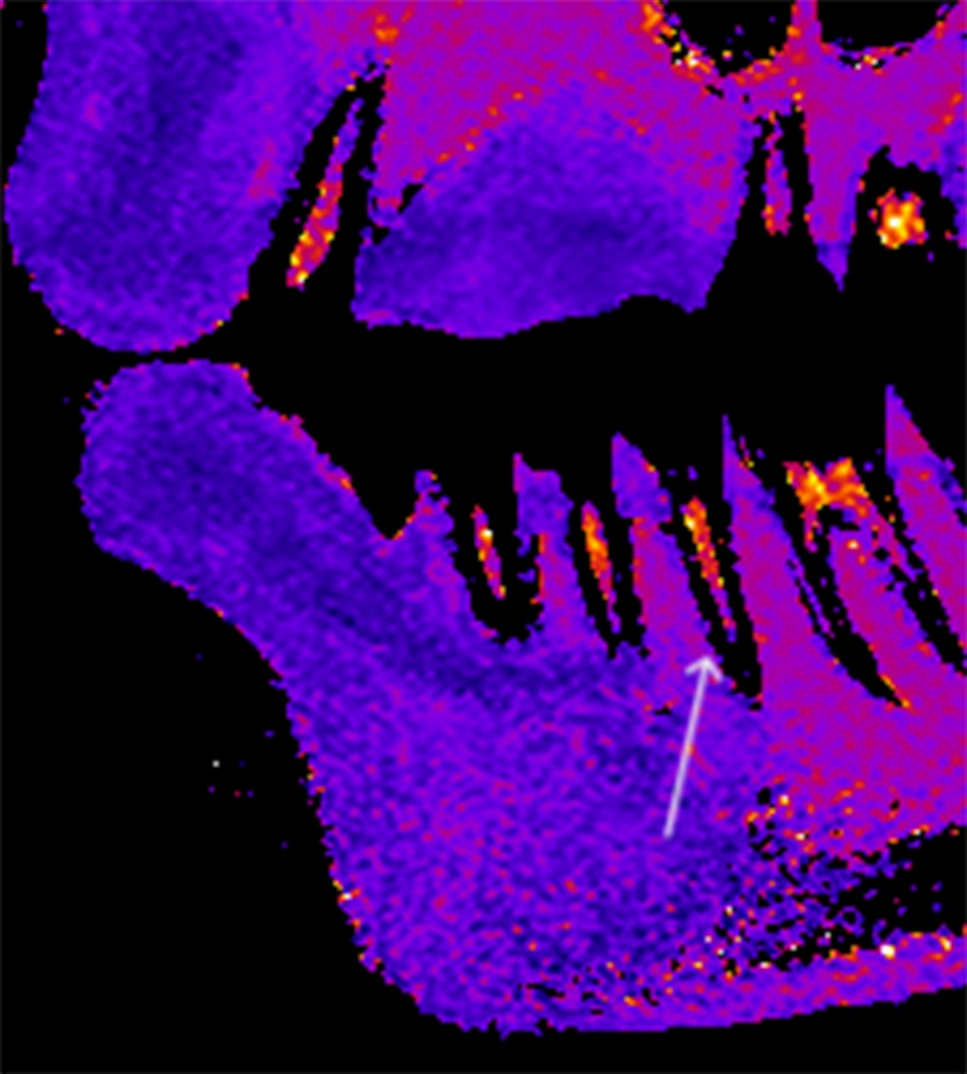
*T*_*2*_ map of a representative patient’s teeth (white arrow) in a sagittal slice

The multi-echo-spin-echo (MESE) images were used to calculate the corresponding maps of *T*_*2*_ relaxation time by using the pixel-wise, mono-exponential, non-negative least-squares fit analysis that is implemented in the MRI Analysis Calculator plugin (ImageJ, National Institutes of Health, USA). Finally, from these a *T*_*2*_ map is calculated [8]. In the next step, *T*_*2*_ values were averaged within each box to reduce the noise. In this step, pixels with zero *T*_*2*_ values, which lay out of the pulp, were excluded from averaging [8]. An example of *T*_*2*_ map of patient’s premolar is presented in Fig. 1.

#### Tissue samples and staining

**Fig. 2:**
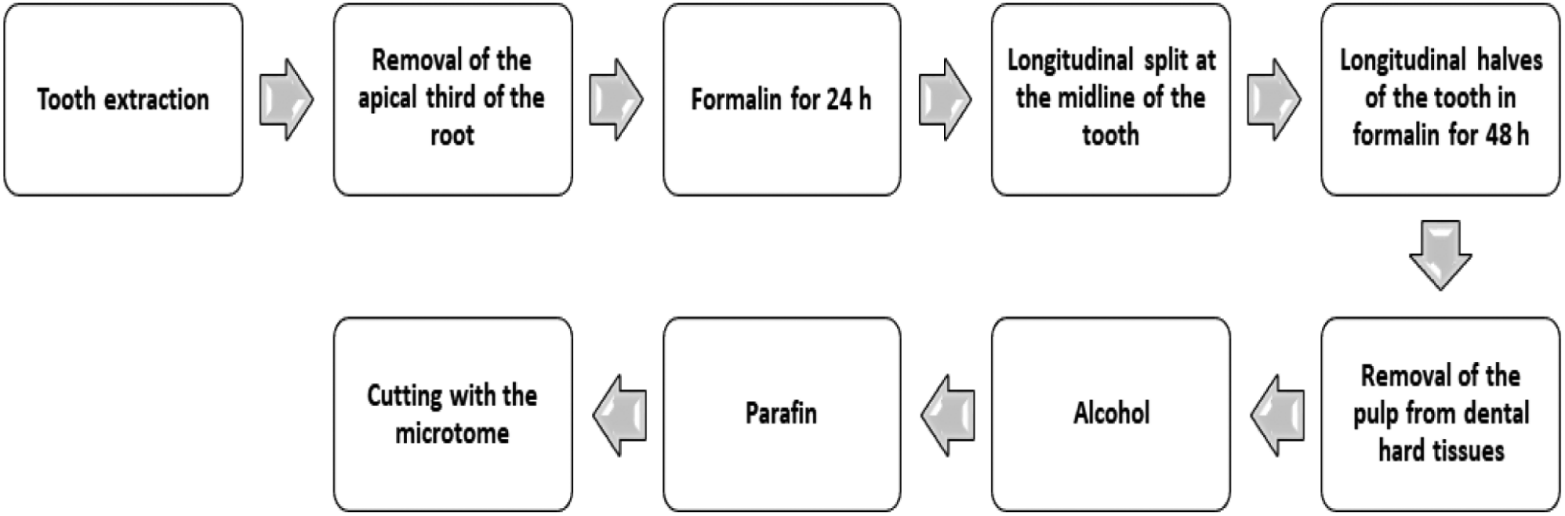
The chart of histological preparation process of the extracted premolars:

The preparation process of the extracted teeth is shown in the Fig. 2 [17].

Immediately after the extraction, one-third of the apical part of the root was cut off for better penetration of the fixating solution into the pulp tissue and fixed in formalin for 24 h [18].

After 24 h, the entire tooth’s vertical (longitudinal) split was done. The halves of the tooth with pulp were re-immersed in formalin for another 48 h [17]. Then, the pulps were gently removed from the dental half, dehydrated in alcohol, immersed in xylene, embedded in paraffin, and cut into 4.5 μm thick longitudinal step serial sections (Fig. 2). The step between the two sections was 20-µm thick [17]. Sections were stored at room temperature and stained with HE. Blood vessels were shown by immunohistochemical labeling of endothelial cells with anti-von Willebrand factor (vWf) (Dako Glostrup, Denmark, 1:800) [19].

For demonstration of inflammatory infiltrate immunohistochemical methods using anti – CD 3 (Dako Glostrup, Denmark, 1:50), anti – CD 68 (Dako Glostrup, Denmark, 1:50) and anti – CD 79a (Dako Glostrup, Denmark, 1:20) were used, former to display T lymphocyte, macrophage cells and latter B lymphocyte.

### Image analysis and evaluation of the volume density of blood vessels **(V**_**vasc.**_**)**

Image analysis was performed under a light microscope (Nikon Eclipse E 400), using a camera (Nikon digital sight DS-M5) and NIS elements version 3 – documentation computer program. The measurements were performed on three sagittal slices of the central part of the dental pulp at the objective magnification of 40x for blood vessels in the whole section (crown and root part) of the pulp tissue [17].

The volume density of the blood vessels lumen was stereologically analyzed, respectively, using the Weibel’s test system described previously [20].

### Image analysis and quantification of inflammatory infiltrate- density of T lymphocyte, macrophage and B lymphocyte infiltrate **(N/mm**^**2**^**)**

Image analysis was performed using the same optical microscope and software as for the analysis of the volume density of blood vessels. The sagittal slices of the central part of the dental pulp were firstly outlined at the objective magnification of 10x, in order to acquire a measurement of the area, which was expressed in mm^2^. Secondly, at the objective magnification of 20x, all cells positive to CD 3 and CD 68, separately, were counted, consequently gaining the density of T lymphocyte and macrophage infiltrate.

All cell countings, including those of inflammatory cells as well as blood vessel cells and MRI *T*_*2*_ relaxation times were performed separately, for the *crown part* and *root part* of the tooth sample.

### Statistics

Mean signal values and variability of *T*_*2*_ relaxation times in distinct regions of interest (ROI) on *T*_*2*_-weighted images were obtained. Statistical analysis was performed using SigmaPlot 14.0 (Systat Software, Inc., USA). Shapiro-Wilk test and Brown-Forsythe were used to check for normality and equal variance. One-way analysis of variance (ANOVA) was used to test for differences between parameters. In cases when Shapiro-Wilk or Brown-Forsythe test failed, Friedman ANOVA on Ranks was performed. If ANOVA showed statistically significant differences between groups of data, Dunnett’s method for multiple comparisons was used. The level was set at 𝑝 < 0.05 for all statistical significances.

The sample size was determined, using the power of the study at 0.8 and *p - value* significance at < 0.01. The result of appropriate sample size was *N* = 19. Our study involved 52 specimens.

The correlation between the volume density of blood vessels, T lymphocyte infiltrate density, B lymphocyte infiltrate density, macrophage density and corresponding *T*_*2*_ relaxation times acquired by MRI, were tested by the Pearson coefficient of correlation (*p* < 0.05).

## Results

Fifty-two upper and lower permanent premolars of fourteen patients (nine female and five male) were included in the study. Mean age was 14,2 ± 1,97 years.

### Findings of caries progression assessment (ICDAS)

Each extracted premolar had at least one initiate carious lesion visible on its surface. When several carious lesions were visible on the same tooth, code with a higher ICDAS score was noted and included in statistic calculation. No grater carious decay than ICDAS 3 were present on extracted teeth. All carious lesions were assessed as inactive type with ceased progression.

### MRI results; T_2_ relaxation times

Mean *T*_*2*_ relaxation times and variability of *T*_*2*_ relaxation times in the groups of teeth from ICDAS 0 to ICDAS 3 are shown in Table 1.

### Qualitative and quantitative findings of tissue samples

#### Pulp vascularity

**Fig. 3:**
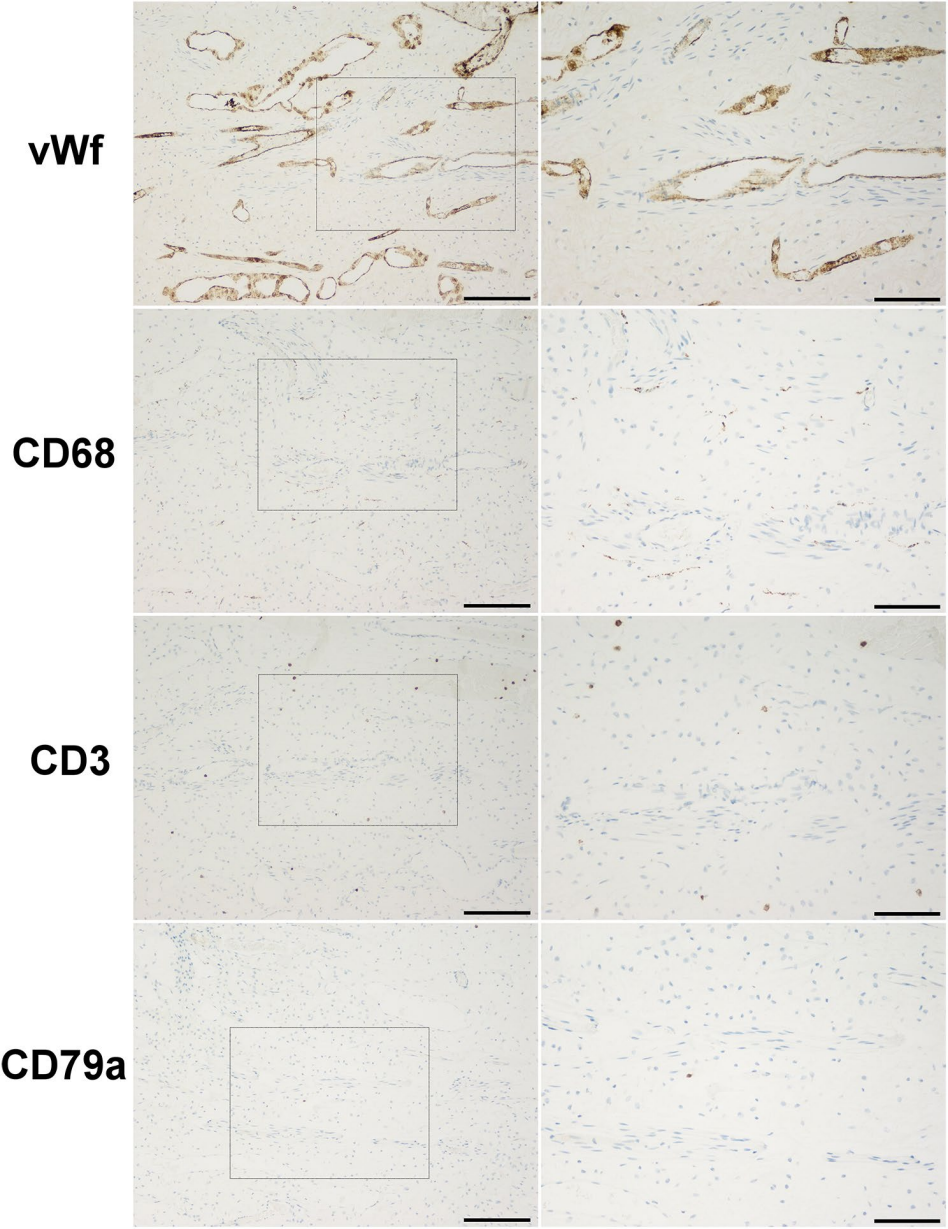
Dental pulp of a lower premolar assessed with ICDAS 2 score, stained with anti- vonWilendbrand factor (vWf), anti – CD68, anti – CD3 and anti – CD79a (objective magnification from left to right: × 2, bar = 1000 μm; × 10, bar = 200 μm; × 20, bar = 100 μm). Note that the target cells are revealed in brown colouration

Histological examination of the pulps stained immunohistochemically for von Willebrand factor showed endothelial cells of blood vessels (Fig. 3). In each specimen, crown part of the pulp tissue contained arterioles with very thin and in few voxels even absent muscular stratum. Towards the odontoblastic layer arterioles presented much more branched appearance, which later formed into capillary nets. Capillary mesh was the densest in the cell free zone (Weil layer). In the radicular part of the pulp tissue, the vascular microstructure was different, represented by only one or few vessels with wider diameter compared to those seen in the crown. These vessels extended parallel to the root axis, but later lengthways toward the crown, they ran perpendicular and became smaller, presenting with lesser diameter. Vascular volume densities are presented in Table 1, grouped according to the level of progression of the carious lesion from ICDAS 0 to ICDAS 3.

#### Inflammatory infiltrate

Out of 52 specimens, two separate samples of each staining with anti – CD 3 and anti – CD 68 for demonstration of T lymphocyte and macrophage infiltrate, respectively, were rinsed off the slides due to technical difficulties associated with small amount of pulp tissue material. Consequently, those samples were not available for inclusion in the statistics, which was therefore performed on the remained 50 samples.

Cell infiltrate in different extent and dispersion was seen in slides of each and every extracted tooth, regardless of the presence or absence of caries lesion at its surface (Fig. 2). Inflammatory infiltrates were mostly localized in the sub - odontoblastic zone, near cell rich zone (Höhl’s layer).

The average density of macrophage, T lymphocyte and B lymphocyte infiltrate, separately, are listed in Table 1.

**Table 1:** Mean *T*_*2*_ relaxation times and variability of *T*_*2*_ relaxation times (as means ± SD), vascular density, macrophage, T lymphocyte and B lymphocyte density (as median (25% percentile, 75% percentile)) in groups of teeth with distinct ICDAS scores

Group (ICDAS)	A (ICDAS 0)	B (ICDAS 1)	C (ICDAS 2)	D (ICDAS 3)	p value
	*N* = 15	*N* = 7	*N* = 13	*N* = 17	
***T***_***2***_ **relaxation times (ms)**	111.9 ± 11.2	132.3 ± 18.5*	124.6 ± 14.8	112. 6 ± 18.2	0.013
***T***_***2***_ **relaxation time variability (ms)**	44.6 ± 4.9	46.6 ± 9.3	39.0 ± 5.5	38.6 ± 6.2†	0.007
**Vascular volume density (mm3/mm3)**	0.219 (0.09; 0.29)	0.301 (0.29; 0.32) *	0.279 (0.19; 0.31)	0.221 (0.16; 0.28) ‡	0.023
**Macrophage density (N/mm** ^**2**^ **)**	5.847 (2.30; 7.18)	19.894 (11.89; 25.92) *	27.500 (7.17; 41.07) ⁑	23.288 (16.15; 61.15) †	< 0.001
**T lymphocyte density (N/mm** ^**2**^ **)**	1.734 (0.71; 3.03)	7.778 (3.99; 12.11)	10.000 (5.52; 13.13) ⁑	5.000 (1.81; 13.40) †	0.001
**B lymphocyte density (N/mm** ^**2**^ **)**	0.000 (0.00; 0.13)	0.160 (0.13; 0.78)	2.240 (0.59; 4.007) ⁑	1.989 (0.81; 9.57) †	< 0.001

*- statistically significant difference between group A (ICDAS 0) and B (ICDAS 1)

⁑- statistically significant difference between group A (ICDAS 0) and C (ICDAS 2)

†- statistically significant difference between group A (ICDAS 0) and D (ICDAS 3)

‡- statistically significant difference between group B (ICDAS 1) and D (ICDAS 3)

### Correlations between measured parameters

A positive correlation was observed between *T*_*2*_ relaxation times and macrophage and T lymphocyte density in healthy teeth (Table 2).

There was a positive correlation between vascular volume density and *T*_*2*_ relaxation times of dental pulp in teeth with ICDAS score 1. In contrast, there was a negative correlation between *T*_*2*_ relaxation times and macrophage density.

There was also a positive correlation between *T*_*2*_ relaxation time variability and macrophage and T lymphocyte density in teeth with ICDAS score 2.

In teeth with ICDAS score 3, a positive correlation between *T*_*2*_ relaxation times and *T*_*2*_ relaxation time variability and lymphocyte B density was observed.

**Table 2:** Correlations between measured parameters in the teeth with distinct ICDAS scores

		Vascular volume density (mm3/mm3)	Macrophage density (N/mm^2^)	T lymphocyte density (N/mm^2^)	B lymphocyte density (N/mm^2^)
Group A (ICDAS 0)	T_2_ relaxation times (ms)	NS	*R* = 0.599 *p* = 0.0397	*R* = 0.741 *p* = 0.00901	NS
	T_2_ relaxation times variability (ms)	NS	NS	NS	NS
**Group B** **(ICDAS 1)**	***T***_***2***_ **relaxation times (ms)**	*R* = 0.812 *p* = 0.0251	*R* = − 0.821 *p* = 0.0253	**NS**	**NS**
	***T***_***2***_ **relaxation times variability (ms)**	**NS**	**NS**	**NS**	**NS**
**Group C** **(ICDAS 2)**	***T***_***2***_ **relaxation times (ms)**	**NS**	**NS**	**NS**	**NS**
	***T***_***2***_ **relaxation times variability (ms)**	**NS**	*R* = 0.598 *p* = 0.04	*R* = 0.620 *p* = 0.0316	**NS**
**Group D** **(ICDAS 3)**	***T***_***2***_ **relaxation times (ms)**	**NS**	**NS**	**NS**	*R* = 0.747 *p* = 0.000875
	***T***_***2***_ **relaxation times variability (ms)**	**NS**	**NS**	**NS**	*R* = 0.634 *p* = 0.00831

## Discussion

Our results show an increase in vascular density and *T*_*2*_ relaxation times in teeth with ICDAS score 1. The inflammatory cells, such as T lymphocyte and macrophages, are present in healthy as well as in the teeth with caries. In latter, the values were significantly higher [3, 5]. In contrast, B lymphocytes appear only in carious teeth [5]. A positive correlation was observed between *T*_*2*_ relaxation times and macrophage and T lymphocyte density in healthy teeth. In teeth with ICDAS score 1, there was a positive correlation between *T*_*2*_ relaxation times and vascular volume density and a negative with macrophage density. There was also a positive correlation between *T*_*2*_ relaxation times and B lymphocyte density in teeth with ICDAS score 3.

We observed the inflammatory cells, such as T lymphocyte and macrophages in healthy as well as in the teeth with caries. These results are in agreement with study of Opasawatchi et al. (2022), where T lymphocyte and macrophages were presented in healthy as well as in the carious teeth, whereas macrophage infiltration was seen in greater extent in carious infected pulps [21]. In addition, with the higher ICDAS score, we observed more pronounced macrophage infiltration along with moderate increase of lymphocyte infiltration. The possible explanation is that the intensity of pulp tissue response to macrophage infiltration increases with the degree of carious lesion and greater permeability of hard dental tissues, as shown in previous studies [21, 22].

Similar as in our study, Tomaszewska et al. performed analysis on non – carious orthodontically extracted upper premolars using immunohistochemical staining of inflammatory cells with anti – CD 68 and blood vessels with anti – CD 31 [23]. Their results showed the presence of inflammatory infiltrate and cell atrophy even in pulps of intact teeth, without any caries or trauma visible on their surface. The mentioned authors suggested inflammation might be caused by the fact that all patients had tooth crowding and malocclusion. According to mentioned study, it is possible the inflammatory infiltrate seen in our histologic slides might as well be due to orthodontic factor and not only due to carious lesion, since our study was also performed on the patient’s teeth with orthodontic diagnosis.

In our study, every lesion was inactive and all participants reported no symptoms, nor pain or discomfort regarding having a carious lesion. Considering this, all pulps were clinically considered to be healthy. In daily clinical practice, dentists are confronted with much more challenging situations, where pulp might be mild, moderately orseverely inflamed, even partially necrotic [24]. The correlation between the clinical status of the pulp and its histological state was assessed in many studies, in which no strong connection of histological pictures and clinical signs were confirmed [24–26].

In the present study, an increase of both, *T*_*2*_ relaxation times and vascular density, in teeth with ICDAS score 1 and their positive correlation was observed. An increase in *T*_*2*_ relaxation times is in agreement with the physiological response of the vascular pulp tissue to infectious carious disease at its early stage [8]. This could be explained with the observed increase in vascularity due to hyperaemia and angiogenesis [27]. In teeth with higher ICDAS scores, the decrease of *T*_*2*_ relaxation times is observed, suggesting that more inflammatory cells infiltration prevails over vascularity in the pulpal tissue. In our study, this was confirmed with the progression of macrophage infiltration in ICDAS scores 2 and 3. Additionally in these, ICDAS scores along with the progression of inflammatory cells infiltration, moderate positive correlations were observed with *T*_*2*_ relaxation time variability. This could be explained by greater tissue inhomogeneity, due to vascular density decease and increase in inflammatory cells infiltration, that progressed from the crown to the root part of the pulpal tissue.

In our study, the B lymphocytes appear only in carious teeth. This is in agreement with the findings of previous studies, which concluded, that there is usually a lack of B lymphocytes in a healthy pulp [5]. In contrast, they infiltrate tissue in a later phase, after inflammation has already been evolved [5]. This could be confirmed by our results. Namely, we observed strong positive the correlation between B lymphocytes density and the *T*_*2*_ relaxation time and its variability in teeth with ICDAS score 3.

Our study has several limitations. Firstly, it must be noted that teeth included in the study were not all from different patients and were therefore in principle not entirely independent. At least 2 and mostly 4 teeth from the same patient were included in the study and observed in one examination in the same field of view. This was due to our efforts to maximize the number of studied samples and due to limited availability of MRI for the study. In addition, average values of *T*_*2*_ relaxation times were lower than those seen in the study of Cankar et al. [8], especially in the group of healthy teeth (ICDAS 0). This could be explained by the age difference. The patients included in the present study are younger and the difference might occur due to the influence of age on the pulpal tissue. Lastly, comparison of *T*_*2*_ relaxation times among different ICDAS scores in the present study is limited due to the fact that our study did not include teeth with deeper and more extensive carious lesion (ICDAS 4, 5 and 6).

## Conclusion

The results of the present study confirm that the difference in the dental pulp tissue between teeth with ICDAS score 0 and teeth with ICDAS score 1 is well presented in *T*_*2*_ relaxation times. In contrast, in teeth with ICDAS score 2 and 3, the progression of inflammation favours positive correlation between *T*_*2*_ relaxation time variability and inflammatory cells infiltration, possibly due to greater tissue inhomogeneity. In teeth with ICDAS score 3, the *T*_*2*_ relaxation time and its variability, correlate with B lymphocytes infiltration.

## Data Availability

The datasets used and analysed during the current study are available from the corresponding author on reasonable request.
